# Construction and validation of an evaluation instrument for the care pathway of people with syphilis

**DOI:** 10.1590/0034-7167-2025-0072

**Published:** 2025-12-08

**Authors:** Rafaela Bezerra Façanha Correia, Harlon França de Menezes, Cristiane da Silva Ramos Marinho, Janmilli da Costa Dantas, Rafaela Prudlik Mourad, Richardson Augusto Rosendo da Silva

**Affiliations:** I Universidade Federal do Rio Grande do Norte. Natal, Rio Grande do Norte, Brazil

**Keywords:** Syphilis, Comprehensive Health Care, Health Services, Health Care Evaluation Mechanisms, Validation Study., Sífilis, Atención Integral de Salud, Servicios de Salud, Evaluación en Salud, Estudio de Validación.

## Abstract

**Objectives:**

to construct and validate the content of an evaluation instrument for the Care Pathway of people with syphilis.

**Methods:**

this methodological study was conducted in four stages: 1) theoretical framework through a scoping review; 2) construction of the instrument; 3) content validation using the Delphi technique; and 4) finalization of the instrument. Data analysis was performed using the Item-Level Content Validity Index, reliability applied to domains, instrument-level content validity, and Cronbach’s alpha coefficient.

**Results:**

the validation of the instrument involved item assessment through two rounds of the Delphi technique. The final version of the instrument consisted of 58 questions distributed across two dimensions: macropolitics and micropolitics of health care. Each item achieved a validity index of 1.00.

**Conclusions:**

the instrument demonstrated content validity and reliability, contributing to improvements in health services, enhanced integration among professionals, and reduced syphilis transmission.

## RESUMEN

**Objetivos:**

construir y validar el contenido de un instrumento para la Evaluación de la Línea de Cuidado a la persona con sífilis.

**Métodos:**

estudio metodológico desarrollado en cuatro etapas: 1) levantamiento teórico mediante una revisión de alcance; 2) construcción del instrumento; 3) validación de contenido mediante la técnica Delphi; y 4) finalización del instrumento. El análisis de los datos se realizó a partir del Índice de Validez de Contenido por ítem, la Fidedignidad aplicada a los Dominios, la Validez de Contenido aplicada al Instrumento y el coeficiente alfa de Cronbach.

**Resultados:**

la validación del instrumento implicó la evaluación de los ítems en dos rondas Delphi. El instrumento quedó constituido por 58 preguntas distribuidas en dos dimensiones: macropolítica y micropolítica del cuidado en salud. Se obtuvieron índices iguales a 1,00 en cada ítem.

**Conclusiones:**

el instrumento demostró validez y confiabilidad de contenido, contribuyendo a la mejora de los servicios de salud, la integración entre profesionales y la reducción de la transmisión de la sífilis.

## INTRODUCTION

Syphilis is a serious public health problem. If left untreated, it can cause severe complications such as neurological, ocular, otologic, and cardiovascular disorders^(1,2)^. In Brazil’s public health system, syphilis control includes health education initiatives, condom distribution, screening through rapid testing, and facilitated access to health services for diagnosis and treatment. However, significant challenges remain in the effective implementation of these measures^(3,4)^, largely due to the fragmentation of care within the Unified Health System (SUS in Portuguese).

Internationally, health system integration has been adopted as one of the main strategies to increase the efficiency of health services. Nevertheless, despite ongoing efforts, addressing fragmentation and delivering more equitable, comprehensive, and accessible solutions remains a major challenge for most countries in the Americas^(5)^.

In an attempt to overcome this fragmentation of care and to ensure a set of quality actions and services, the SUS has pursued the reorganization of the Health Care Network (RAS in Portuguese) as a way to strengthen the principle of comprehensiveness. As a guiding approach, the organization and operationalization of Care Pathways (CP) have been adopted^(6)^.

The Care Line (LC) establishes an assistive pathway for the user across different points of attention within the Health Care Network (RAS), integrating preventive, diagnostic, and treatment actions to ensure comprehensive and longitudinal care. Its structure outlines possible itineraries that the user may follow based on their specific health needs^(7)^.

Given this context, health professionals, social control bodies, and municipal, state, and federal managoers face the challenge of implementing, monitoring, and evaluating the proposed strategies. To this end, the development and validation of a reliable instrument to evaluate the care pathway for individuals with syphilis is necessary.

Thus, this research becomes highly relevant as it introduces a new technology for health management, strengthening public policies aimed at syphilis control and improving the workflow for diagnosis, treatment, follow-up, and prevention of affected individuals. By enhancing these processes, the study contributes to a more integrated model of care.

Furthermore, this instrument has the potential to improve the care pathway for individuals with syphilis, demonstrating both its practical impact and its ability to guide future health interventions. It also holds significant value for public health and nursing by supporting new studies in the field of Health Management and Planning within the Brazilian Unified Health System (SUS

## OBJECTIVES

To develop and validate an evaluation instrument for the care pathway of people with syphilis.

## METHODS

### Ethical Aspects

This study was approved by the Research Ethics Committee. The research complied with Resolution No. 466/12 of the National Health Council, and all participants signed the Informed Consent Form (ICF).

### Study Design, Period, and Phases

This is a methodological study conducted from January to December 2024 and developed in four phases: 1) theoretical framework through a scoping review; 2) instrument construction; 3) content validation using the Delphi technique; and 4) finalization of the instrument.

The SQUIRE 2.0 (Standards for Quality Improvement Reporting Excellence) guidelines were followed to enhance quality standards and ensure methodological rigor in publications.

### Population, Sample, and Inclusion and Exclusion Criteria

For the third phase-content validation-experts were selected through the Lattes Platform of the National Council for Scientific and Technological Development (CNPq in Portuguese), using an advanced subject search with the term “syphilis.” The following filters were applied: Academic Background/Degree: All; Country: Brazil; Region/State: All; Professional Activity: Major Area: Health Sciences; Area: All; Subarea: All; Specialty: All. The initial search identified 125 nurses.

The inclusion criteria for selecting specialists in this study were: holding a master’s or doctoral degree; being a researcher, professor, or health professional; working in one of the following fields: syphilis, evaluation of health services and systems; having an updated Lattes curriculum within the last 12 months; and having published studies or articles on the subject in the last five years.

For sample size calculation, the following formula was applied: n = Z^2^ 1- α/2. p.(1-p) / n2, where: “Z^2^ 1-α/2” **=** confidence level adopted; “p” **=** expected proportion of specialists; and “e” **=** acceptable proportional difference from the expected. A 95% confidence level was set (Z^2^ 1-α/2 = 1,96), with an expected proportion of 85% specialists and a sampling error of 15%, leading to an ideal sample size of 22 specialists^(8)^. Considering the challenges in obtaining responses in validation studies, a larger sample was invited. Thus, 75 experts were contacted to participate as judges in the content validation process, ensuring a robust assessment and reliability in the findings.

### Study Protocol

To identify the constitutive elements of the evaluation instrument for the care pathway of individuals with syphilis, a Scoping Review (SR) was conducted in the first phase, following the six recommended steps: identifying the research questions; identifying relevant studies; selecting the studies; mapping and extracting the results; and narratively synthesizing the findings^(9)^.

The formulation of the guiding research question followed the PCC strategy (Population - Concept - Context), structured as follows: Population - individuals with syphilis; Concept - evaluation of the care pathway; Context - macroand micropolitical dimensions of healthcare delivery. Based on this framework, the following research question was established: “What elements should be included in an evaluation instrument for the care pathway of individuals with syphilis within the macroand micropolitical dimensions of healthcare?”.

The literature search was conducted between January and February 2024 and divided into three phases, aiming to identify primary studies, unpublished literature (gray literature), and systematic reviews. The initial search was performed across multiple databases, including PubMed^®^, Web of Science, Scopus, CINAHL, Cochrane Library, Scientific Electronic Library Online (SciELO), and Latin American and Caribbean Health Sciences Literature (LILACS). The search utilized indexed descriptors (Health Sciences Descriptors - DeCS and Medical Subject Headings - MeSH), combined with Boolean operators: Syphilis; Health Evaluation; Health Services; Delivery of Health Care; Public Health Practice; and Health Services Administration.

A complementary search was conducted using Google Scholar^®^, incorporating index terms and synonyms (keywords) identified in the first phase, including “care pathway,” “macropolitical dimension,” “micropolitical dimension,” “evaluation instrument,” and “individuals with syphilis.” A reverse search was performed, analyzing references cited in studies selected from the previous phases. This three-step scoping review approach enables the identification of additional materials or research not retrieved from traditional databases^(9)^.

The search and selection of records were conducted by two doctoral candidates in Nursing, following a predefined protocol created and registered on the Open Science Framework (OSF) platform (identifier: 10.17605/OSF.IO/KPSAB). Discrepancies were resolved through mutual consensus. The study selection began with a floating reading of titles and abstracts, applying predefined eligibility criteria. Records meeting the inclusion criteria advanced to full-text review for final inclusion in the study sample.

The eligibility criteria were defined based on the research questions, including primary studies addressing elements for the construction of an evaluation instrument for the care pathway of individuals with syphilis within the macropolitical and micropolitical dimensions of healthcare. Studies were included if they were published in Portuguese, English, or Spanish, with no time restrictions. Exclusion criteria encompassed letters to the editor, editorials, review articles, single-case studies, abstracts, conference proceedings, books, protocols, undergraduate theses, dissertations, and articles not available in full text.

Data collection was guided by an original protocol, which allowed for the organization and tabulation of information using Microsoft Excel, version 2013. The extracted data included authors, year of publication, country of origin, language, journal name, journal scope, database, study design, level of evidence, study objectives, attributes, antecedents, consequences, additional concepts, and study findings. These data provided a comprehensive and cohesive understanding of the reviewed studies, enabling a rigorous and detailed evaluation of the literature.

Initially, 5,622 records were identified through database searches. After removing duplicates, ineligible records, and those that did not meet the inclusion criteria, 5,322 records underwent title and abstract screening, resulting in the exclusion of 5,329 records. Subsequently, 115 full-text articles were assessed for eligibility, of which 84 were excluded based on predefined criteria. Ultimately, 31 studies were analyzed.

From this analysis, key evaluation themes emerged for the development of the assessment instrument. The following thematic units were defined: Educommunication; Enhancement of Strategic Information; Expansion of Investigation Committees for the Prevention of Vertical Transmission of HIV, Syphilis, and Viral Hepatitis; Strengthening of Healthcare Networks; Reinforcement of Partnerships Between the Ministry of Health and Other Stakeholders; Rapid Response to Syphilis Within Healthcare Networks. Based on this thematic categorization, the instrument was structured according to the care pathway framework, considering both microand macropolitical healthcare management perspectives^(10)^.

The process of constructing the instrument initially involved defining the items that would comprise it, based on the scoping review. The analysis was guided by the care pathway framework from both micropolitical and macropolitical perspectives, as well as the Strategic Agenda for the Reduction of Syphilis in Brazil^(11)^ and the Clinical Protocol and Therapeutic Guidelines for Syphilis Prevention^(12)^. Based on the scoping review results, the main items were selected to form the instrument.

Thus, the preliminary construction of the instrument began. Meetings were held with four expert researchers with knowledge of syphilis to analyze the previously drafted questions and response options. After discussion, the items were revised or retained according to each researcher’s recommendations, reaching a consensus on the minimum number of selected categories. The language used was designed to ensure clear, simple, and objective communication, avoiding ambiguity in interpretation. After consensus was reached between the researchers and the authors, the instrument consisted of 74 items.

In the third phase, content validation, healthcare professionals participated using the Delphi technique, which required two rounds to reach consensus. Contact was made via email, including an invitation letter, the ICF, and a link to the electronic questionnaire hosted on SurveyMonkey. A 20-day deadline was established for each evaluator to accept the ICF and complete the instrument, starting from the date the email was sent.

The application of the forms took place between February and April 2024. Invitees who did not complete the instrument evaluation within the established deadline were excluded from the sample. Judges were asked to assess each item based on the criteria of clarity, comprehension, language, and relevance to verify the scope and representativeness of the instrument’s domains. Additionally, they could suggest removals, additions, or modifications to the items.

In the constructed instrument, specialists indicated their level of agreement by marking an “x” on a Likert-type scale, which included the following response options: 1) Not relevant; 2) Slightly relevant; 3) Relevant; and 4) Highly relevant. Thus, 29 specialists participated in the first round, while 22 specialists participated in the second round. The majority were male, primarily from the academic field, and held doctoral degrees.

In the final stage, the data collection instrument was formatted into its final version. The instrument was once again presented to the judges so they could confirm and endorse the changes made, ensuring its accuracy and applicability. After this final review, the instrument was officially formatted, and all judges approved it as the version to be applied in the study.

### Data Analysis

The data obtained were analyzed based on the assessment of the responses collected from the study participants. The information gathered was stored and processed in computerized databases using Microsoft Office Excel and Statistical Package for the Social Sciences (SPSS), version 22.0.

To assess the internal consistency of the instrument (analytical procedures), the Item-Level Content Validity Index (I-CVI), the Domain-Level Reliability Index (DLRI), and the Instrument-Level Content Validity Index (I-CVI) were calculated^(13)^, based on the simple frequency of expert responses. Accordingly, only items with agreement rates equal to or greater than 80% (0.80) were retained in the instrument (across both rounds).

At the end of each item under evaluation, there was a specific field for open-ended responses, allowing expert judges to justify their ratings and/or suggest improvements to enhance the instrument’s relevance. The index score was calculated by summing the number of responses rated as (3) or (4) by the experts. The formula for calculating each item’s validity was: I-CVI = number of (3) + (4) responses / total number of responses.

Moreover, to analyze the consistency and reliability of the instrument, Cronbach’s alpha coefficient was calculated. This test is one of the most widely used measures in research involving the development and application of assessments, allowing the verification of correlations between questionnaire responses. For the Cronbach’s alpha analysis, a threshold of ≥ 0.70 was adopted, which is considered acceptable for exploratory studies and provides evidence of an internally consistent scale.

## RESULTS

The initial version of the instrument contained 74 items; the final version was structured with 58 items: 49 in Dimension 1 - Macropolitics of Health Care, and 9 in Dimension 2 - Micropolitics of Health Care.

The content validation process was conducted in two rounds (Delphi 1 and Delphi 2). Based on the evaluations from the first Delphi round, some questions were excluded or modified. Of the items in the first version of the instrument, 16 (21.62%) were not stabilized in the first round, and 18 (24.32%) had suggestions for wording reformulation. At the end of Delphi 2, the Item-Level Content Validity Index (IVC-I) reached 1.00 for each item, and the overall Content Validity Index (IVC) was also 1.00. Subsequently, minor adjustments were made, and the instrument was finalized with 58 items, thus completing the content validation phase. Regarding the Inter-Rater Validity Index (IVI), the instrument scored 0.84 in Delphi 1 and 1.00 in Delphi 2.

Through Cronbach’s Alpha, the data reliability was confirmed. It was observed that all domains evaluated by specialists obtained an alpha above 0.80. From a dimensional perspective, Phase 2 yielded better overall results compared to Phase 1; however, both phases demonstrated highly satisfactory reliability, as presented in Table 1.

**Table 1:** Data reliability concerning items and dimensions of the Care Pathway Evaluation Instrument for individuals with syphilis, Natal, Rio Grande do Norte, Brazil, 2024

Dimensions	Cronbach's Alpha	
	Phase 1	Phase 2
Dimension 1 - Macropolitical Health Care	0.865	0.984
Dimension 2 - Micropolitical Health Care	0.818	0.977
Overall Evaluation	0.825	0.972

Chart 1 presents the final version of the Evaluation Instrument for the Care Pathway of People with Syphilis, as well as the I-CVI for each item and the DLRI for the domains.

**Chart 1:** Evaluation Instrument for the Care Pathway of People with Syphilis - Final Version, Natal, Rio Grande do Norte, Brazil, 2024

Dimension 1 - Macropolitics of Health Care		I-CVI	DRI
01	Is the Care Pathway for People with Syphilis included in the State Health Plan?	1.00	1.00
02	Does the Care Pathway for People with Syphilis organize the flow and counterflow of individuals within Primary Health Care (PHC), support systems, and secondary and tertiary care services?	1.00	
03	Does the Care Pathway for People with Syphilis provide updated clinical protocols and therapeutic guidelines for exposed children and congenital syphilis, for pregnant women with syphilis, and for acquired syphilis?	1.00	
04	Do health services operate with Information Systems to record and monitor process indicators and provide clinical, epidemiological, and management information on syphilis?	1.00	
05	Is the annual preparation and dissemination of syphilis epidemiological data carried out?	1.00	
06	Are acquired, congenital, and gestational syphilis cases investigated and reported by health services, including clinical and laboratory follow-up and case closure?	1.00	
07	Does the Care Pathway for People with Syphilis establish integration among specialized services, testing and counseling centers, hospitals, maternity units, and PHC?	1.00	
08	Is PHC the main entry point for syphilis care, focusing on health promotion, prevention, diagnosis, treatment, and control of syphilis?	1.00	
09	In PHC, does each team member have defined skills and competencies for syphilis care?	1.00	1.00
10	Is PHC organized to provide appropriate care to pregnant women with syphilis and their partners, according to Ministry of Health recommendations?	1.00	
11	Does PHC monitor children exposed to syphilis and with congenital syphilis?	1.00	
12	Does PHC offer rapid testing for syphilis during all hours of operation?	1.00	
13	Does PHC offer non-treponemal testing for post-treatment monitoring (cure control)?	1.00	
14	Does PHC administer benzathine benzylpenicillin?	1.00	
15	Do PHC referrals to other health services include information about previous management and test results for the person with syphilis?	1.00	
16	Are there specialized outpatient reference services for individuals with acquired syphilis?	1.00	
17	Do children with congenital syphilis have guaranteed access every six months, for two years, to ophthalmologic consultations?	1.00	
18	Do children with congenital syphilis have guaranteed access every six months, for two years, to audiological exams?	1.00	
19	Do children with congenital syphilis have guaranteed access every six months, for two years, to neurological consultations?	1.00	
20	Do children with congenital syphilis have guaranteed access to treponemal testing (after 18 months of age), when necessary?	1.00	
21	Do children with congenital syphilis have guaranteed access to complete blood counts, when necessary?	1.00	
22	Do children with congenital syphilis have guaranteed access to tests for liver, pancreatic, renal function, and electrolyte disturbances when necessary?	1.00	
23	Are there secondary care services for the diagnostic workup and investigation of neurosyphilis?	1.00	
24	Are there secondary care services for lumbar puncture, when necessary?	1.00	
25	Are there secondary care services for the treatment of neurosyphilis?	1.00	
26	Do individuals treated for neurosyphilis have access to follow-up lumbar puncture?	1.00	
27	Is treatment available in maternity hospitals/birth centers for pregnant women diagnosed with syphilis?	1.00	
28	Do newborns diagnosed with congenital syphilis have guaranteed access to complete blood counts in maternity settings?	1.00	
29	Do newborns diagnosed with congenital syphilis have guaranteed access in maternity settings to tests for liver, pancreatic, renal function, and electrolyte disturbances?	1.00	
30	Do newborns diagnosed with congenital syphilis have guaranteed access to cerebrospinal fluid (CSF) testing in maternity units?	1.00	
31	Do newborns diagnosed with congenital syphilis have guaranteed access in maternity to long bone radiographs?	1.00	
32	Do newborns diagnosed with congenital syphilis have guaranteed access in maternity to chest radiographs?	1.00	
33	Are there tertiary care services for the diagnosis of neurosyphilis?	1.00	
34	Is there an effective counter-referral instrument?	1.00	
35	Does the pharmaceutical services system include benzathine benzylpenicillin for syphilis treatment?	1.00	
36	Does the pharmaceutical services system include procaine benzylpenicillin for congenital syphilis treatment?	1.00	
37	Does the pharmaceutical services system include crystalline (potassium) benzylpenicillin for neurosyphilis treatment?	1.00	
38	Do health units under the pharmaceutical services system always have sufficient stock of medications for syphilis treatment?	1.00	
39	Does the pharmaceutical services system include pharmacovigilance in syphilis care?	1.00	
40	Does care management include continuing education for professionals involved in syphilis care?	1.00	
41	Are continuing education activities conducted on-site during protected time (working hours)?	1.00	1.00
42	Does continuing education use active learning methodologies and promote meaningful, critical learning?	1.00	
43	Does continuing education occur throughout the institutional career of professionals involved in syphilis care?	1.00	
44	Is there integration between teaching, service, and community?	1.00	
45	Do services within the Care Pathway for People with Syphilis provide condoms as part of routine care?	1.00	
46	Are educational and informational materials on syphilis prevention, diagnosis, treatment, and surveillance developed and disseminated for the general population, key populations, priority populations, among others?	1.00	
47	Are there partnerships among federal, state, and municipal managers, health professionals, the community, and other stakeholders involved in syphilis prevention?	1.00	
48	Are financial resources available for the organization of the Care Pathway for People with Syphilis?	1.00	
49	Is the Care Pathway for People with Syphilis monitored and evaluated?	1.00	
**Dimension 2 - Micropolitics of Health Care**		**I-CVI**	**DLRI**
50	Do PHC teams recognize their role in the care of individuals with syphilis?	1.00	1.00
51	In PHC services, are users cared for by the same physician and/or nurse?	1.00	
52	Does the PHC team develop the Singular Therapeutic Project jointly with the individual with syphilis?	1.00	
53	In PHC, is the user's medical record available during appointments?	1.00	
54	Do PHC professionals integrate and include the topic of syphilis in various community settings (e.g., schools and associations) and within the health service itself (e.g., prenatal groups, elderly groups, waiting rooms, during consultations)?	1.00	
55	Does pharmaceutical care monitor whether the user has completed syphilis treatment?	1.00	
56	Does the pharmaceutical services system include pharmaceutical care for the syphilis-affected population, understood as pharmacist-led actions to guide and monitor appropriate medication use?	1.00	
57	During team meetings, do health teams engage in planning, monitoring, and evaluating the care provided to people with syphilis?	1.00	
58	Does the planning, monitoring, and evaluation of syphilis prevention and control actions include the organized participation of the community?	1.00	

*IVC-I - Índice de Validade de Conteúdo por Item; IFD - Índice de Fidedignidade do Domínio; APS - Atenção Primária à Saúde; LCR - Líquido Cefalorraquidiano.*

## DISCUSSION

This study developed an instrument designed to rigorously assess various aspects related to both the macropolitical and micropolitical dimensions of health care, enabling the evaluation of the operationalization of the CP for individuals with syphilis. Its application may contribute to the planning and enhancement of syphilis-related care and services within the RAS. It may be used by SUS managers, multidisciplinary teams across different RAS services, and researchers from various institutions.

Despite government efforts in recent years, Brazil remains far from eliminating congenital syphilis and from controlling acquired syphilis and syphilis during pregnancy. One of the major challenges facing the SUS continues to be the fragmentation of care, which creates multiple barriers to population access to health services. From the CP perspective, this fragmentation may result from the lack of disease prevention and health promotion actions; the absence of management and regulation of macroprocesses; deficiencies in service networks; lack of caregiver accountability; insufficient supplies; questionable service quality; and difficulties in user access to care^(10)^.

A health system based on comprehensiveness should ensure continuity of care by coordinating a responsible care program from primary health care (PHC) through specialized outpatient clinics to emergency care in hospitals and maternity wards^(14)^. In this regard, urgent initiatives and new technologies are needed to strengthen health system management, particularly with regard to the integration of services from both micropolitical and macropolitical perspectives.

From the macropolitical perspective-i.e., the management level-the implementation of a Care Pathway is proposed^(10)^. For effective implementation, it is necessary to organize health surveillance and information systems, health communication, intersectoral actions, the establishment of specific policies with defined funding, the structuring of the service network, and the adoption of clinical protocols. From the micropolitical perspective-i.e., the level of work processes-the same authors emphasize teamwork in care coordination, the establishment of caregiver-user bonds and responsibility, and the promotion of user autonomy.

Regarding macropolitics, the first dimension of the study instrument aims to organize and structure the Care Line (LC) within public health policies, emphasizing the importance of incorporating LC for individuals with syphilis into the State Health Plan. This inclusion facilitates the integration of this condition into healthcare policies, ensuring coordination among different levels of care. Furthermore, by aligning the flows and counterflows between Primary, Secondary, and Tertiary Care services, the continuity of care is reinforced, enabling proper follow-up for affected individuals^(15)^.

In Brazil, the primary entry point for the diagnosis and treatment of syphilis is Primary Health Care (PHC). Therefore, these services must be accessible, both geographically and functionally, considering factors such as operating hours, availability of rapid testing, and antibiotic treatment^(16)^. Additionally, there is a need for planning, continuous monitoring of actions in territories, and well-trained professionals to effectively manage syphilis cases with qualified listening, free from judgment and prejudice.

Health information systems are crucial for syphilis surveillance, supporting decision-making in promotion, control, and transparency of public health actions^(17)^. To generate reliable data, it is essential to ensure quality, minimize underreporting, and raise awareness among health professionals^(18)^. Thus, data collection and dissemination, along with case notification, allow for local strategy adjustments, facilitating tracking, treatment, and prevention of disease spread. The focus remains on early notification, ensuring immediate control measures and adequate clinical follow-up^(19)^.

The management of syphilis in pregnant women and their partners is crucial for preventing congenital syphilis, helping to reduce maternal and fetal complications and preventing reinfection through preventive actions. Active partner tracing for these pregnant women is an essential strategy, as it facilitates diagnosis, treatment, and strengthens prenatal care for the partner within health services^(20)^.

It is important to note that partners may be infected even if serological tests are non-reactive, requiring adequate treatment and reinforcing the need for health professionals to be properly trained to handle such cases effectively.

Another key target group in Primary Health Care (PHC) is children, with the Ministry of Health recommending that all infants exposed to congenital syphilis during pregnancy receive follow-up care, including monthly outpatient visits until six months of age and bimonthly visits from six to eighteen months, even when their mothers have been properly treated.

The purpose of this monitoring is to identify and treat possible cases of congenital syphilis. Adequate follow-up is fundamental to prevent complications, late sequelae, and ensure healthy child development^(21)^.

It is important to emphasize that effective syphilis treatment involves the entire Health Care Network (RAS). Primary Health Care (PHC) referrals must include complete information to ensure continuity and quality of care^(22,23)^. Specialized services are essential for the diagnosis and management of severe complications, such as neurosyphilis, which requires secondary care due to its complexity and is vital for comprehensive care^(24)^.

Several municipalities in Brazil, including Joinville, Paraná, São Paulo, Belo Horizonte, and Rio de Janeiro, have implemented the Care Pathway for individuals with syphilis. However, they face challenges in assessing and monitoring its effectiveness. The diversity of control actions and regional differences in a country of continental dimensions hinder the development of universally applicable instruments and the validation of their construct and applicability^(25)^. Nevertheless, the validation of these instruments can contribute to process improvements, service qualification, and the implementation of care models aligned with the population’s needs.

The study focused on the development and validation of an evaluation instrument for the care pathway of individuals with syphilis demonstrates significant impacts on nursing practice, particularly within public health. Among the highlighted benefits is the enhancement of clinical practice, enabling nurses to utilize an objective tool for assessing and monitoring the quality of care, facilitating the identification of gaps and the implementation of improvements in service delivery.

Moreover, the strengthening of nurses’ strategic role as leaders in syphilis management is a key contribution. The application of the validated instrument reinforces integrated and effective actions, highlighting the importance of these professionals in managing public health conditions. The use of validated instruments further supports evidence-based decision-making, promoting effective and personalized interventions and optimizing patient outcomes.

Finally, this study has a direct impact on academic training and scientific research, generating relevant knowledge that can be incorporated into the education of future nurses and into studies aimed at the continuous improvement of care for individuals with syphilis.

### Study limitations

One of the limitations of this study is the subjectivity of the expert judges in interpreting the instrument’s questions. It is worth noting that, since the instrument validation was not conducted in real clinical settings, it may not fully reflect the challenges and dynamics encountered in the daily operations of healthcare services.

### Contributions to Nursing, Health, and Public Policy

This new tool for SUS management may support the evaluation and monitoring of services offered to the community. It may also serve as a foundation for the development of strategic actions to control syphilis, contributing to improved care for individuals with this infection, in alignment with the principle of comprehensiveness in health services.

Nursing plays a key role in coordinating care and implementing integrated practices that address the needs of individuals with syphilis, directly aligning with the research objective of developing and validating the Care Pathway Evaluation Instrument. This instrument provides nurses with a robust tool for monitoring care quality, identifying gaps, and promoting improvements in healthcare delivery. Furthermore, by integrating nursing’s strategic role in health policy management and planning, the study strengthens the scientific and practice.

## CONCLUSIONS

The Evaluation Instrument for the Care Pathway of People with Syphilis, validated by experts, consists of 58 questions distributed across the macropolitical and micropolitical dimensions of health care, demonstrating satisfactory validity parameters. Its application in the evaluation of healthcare services can lead to improvements in user accessibility and greater integration between services and professionals, thereby enhancing care for individuals with syphilis and demonstrating the importance of this research.

It is expected that, with a high-quality Care Pathway, transmission rates will decrease, resulting in significant changes in the epidemiological profile in Brazil

## Data Availability

The research data are available within the article.
