# Supplementary material for: Construction and validation of an evaluation instrument for the care pathway of people with syphilis
Source: Rev Bras Enferm. 2025 Dec 8;78(5):e20250072. doi: 10.1590/0034-7167-2025-0072 (PMC12707528; doi:10.1590/0034-7167-2025-0072)
Supplement: Translation

# Translation: Construção e validação de instrumento de avaliação da linha de cuidado à pessoa com sífilis

**DOI:** 10.1590/0034-7167-2025-0072pt

**Authors:** Rafaela Bezerra Façanha Correia, Harlon França de Menezes, Cristiane da Silva Ramos Marinho, Janmilli da Costa Dantas, Rafaela Prudlik Mourad, Richardson Augusto Rosendo da Silva

**Affiliations:** I Universidade Federal do Rio Grande do Norte. Natal, Rio Grande do Norte, Brasil

**Keywords:** Sífilis, Assistência Integral à, Saúde, Serviços de Saúde, Avaliação em Saúde, Estudo de Validação

## Abstract

**Objetivos:**

construir e validar o conteúdo de um instrumento de Avaliação da Linha de Cuidado à pessoa com sífilis.

**Métodos:**

estudo metodológico desenvolvido em quatro etapas: 1) levantamento teórico por meio de revisão de escopo; 2) construção do instrumento; 3) validação de conteúdo pela técnica Delphi, e 4) finalização do instrumento. A análise dos dados foi realizada a partir do Índice de Validade de Conteúdo por item, Fidedignidade aplicado aos Domínios, Validade de Conteúdo aplicado ao Instrumento e coeficiente de alpha de Cronbach.

**Resultados:**

a validação do instrumento envolveu a avaliação dos itens em 2 rodadas de Delphi. O instrumento ficou constituído por 58 questões distribuídas em 2 dimensões: macropolítica e micropolítica do cuidado em saúde. Foram obtidos índices iguais a 1,00 em cada item.

**Conclusões:**

o instrumento demonstrou validade e confiabilidade de conteúdo, contribuindo para melhorias nos serviços de saúde, integração entre profissionais e redução das transmissões de sífilis.

## INTRODUCTION

A sífilis é um grave problema de saúde pública. Se não for tratada, pode causar sérias complicações, como: alterações neurológicas, oculares, otológicas e cardiovasculares^(1,2)^. No sistema de saúde pública do Brasil, o controle da sífilis inclui ações de educação em saúde, oferta de preservativos, rastreamento por meio da testagem rápida e acesso facilitado aos serviços de saúde para diagnóstico e tratamento. Porém, enfrentam-se desafios significativos na implementação efetiva dessas medidas^(3,4)^, resultado de uma fragmentação do cuidado no Sistema Único de Saúde (SUS).

Internacionalmente, a integração do sistema de saúde está sendo utilizada como uma das principais estratégias para aumentar a eficiência dos serviços de saúde. Porém, apesar das tentativas, combater a fragmentação e fornecer soluções mais equitativas, abrangentes e com maior acessibilidade continua a ser um grande desafio para a maioria dos países das Américas^(5)^.

Na tentativa de superar essa fragmentação da atenção e de garantir um conjunto de ações e serviços de qualidade, tem-se buscado no SUS a reorganização da Rede de Atenção à Saúde (RAS) como forma de fortalecer o princípio da integralidade. Como orientação de base, tem-se a organização e a operacionalização das Linhas de Cuidado (LC)^(6)^.

A LC estabelece um percurso assistencial do usuário nos diferentes pontos de atenção da RAS, englobando ações preventivas, diagnósticas e de tratamento, visando alcançar o cuidado integral e longitudinal. O seu desenho prevê possíveis itinerários que o usuário percorrerá, conforme suas necessidades de saúde^(7)^.

Diante dessa realidade, os profissionais de saúde, o controle social, os gestores municipais, estaduais e federal têm o desafio de implantar, acompanhar e avaliar o que está sendo proposto. Para tal, faz-se necessária a construção e validação de um instrumento confiável que permita avaliar a LC à pessoa com sífilis.

Com isso, esta pesquisa torna-se relevante por ser uma nova tecnologia para a gestão em saúde, possibilitando o fortalecimento das políticas públicas direcionadas ao controle da sífilis, além do aperfeiçoamento do processo de trabalho para a melhoria da qualidade do diagnóstico, tratamento, acompanhamento e prevenção às pessoas com esse agravo e assim, contribuir para o cuidado mais integrado. Ademais, este instrumento poderá contribuir para a melhoria da linha de cuidado à pessoa com sífilis, evidenciando tanto o impacto prático quanto o potencial para orientar futuras intervenções no âmbito da saúde pública e enfermagem, ao subsidiar novos estudos na linha de pesquisa de Gestão e Planejamento em Saúde no SUS.

## OBJETIVOS

Construir e validar um instrumento de avaliação da linha de cuidado à pessoa com sífilis.

## MÉTODOS

### Aspectos Éticos

Este estudo foi aprovado pelo Comitê de Ética em Pesquisa (CEP). A pesquisa seguiu a Resolução 466/12 do Conselho Nacional de Saúde e todos os participantes assinaram o Termo de Consentimento Livre e Esclarecido (TCLE).

### Desenho, período e etapas do estudo

Trata-se de um estudo metodológico, realizado no período de janeiro a dezembro de 2024, e desenvolvido em quatro etapas: 1) levantamento teórico por meio de revisão de escopo; 2) construção do instrumento; 3) validação de conteúdo pela técnica Delphi, e 4) finalização do instrumento.

Foram seguidas as diretrizes SQUIRE (*Standards for QUality Improvement Reporting Excellence*) 2.0 para melhoria dos padrões de qualidade e diretrizes metodológicas para as publicações.

### População, amostra e critérios de inclusão e exclusão

Para a realização da terceira etapa, a validação de conteúdo, participaram especialistas selecionados por meio da Plataforma Lattes do Conselho Nacional de Desenvolvimento Científico e Tecnológico (CNPq), utilizando-se a busca avançada por assunto com o termo “sífilis”. Foram utilizados os seguintes filtros: Formação Acadêmica/Titulação: Todas; País: Brasil; Região/UF: Todas; Atuação Profissional: Grande Área: Ciências da Saúde; Área: Todas; Subárea: Todas; Especialidade: Todas. A busca inicial foi composta por 125 enfermeiros.

Como critérios de inclusão, considerou-se os seguintes: ter mestrado ou doutorado; ser pesquisador, docente ou profissional da área da saúde; e trabalhar com uma das seguintes temáticas: sífilis, avaliação em serviços e sistemas de saúde; possuir currículo lattes atualizado nos últimos 12 meses; e ter publicado trabalhos ou artigos sobre a temática nos últimos 5 anos.

Para o cálculo da amostra de especialistas, foi utilizada a seguinte fórmula: n = Z^2^ 1- α/2. p.(1-p) / n2, onde “Z^2^ 1-α/2” = nível de confiança adotado; “p” = proporção esperada dos especialistas; e “e” = diferença de proporção aceitável em relação ao que seria esperado. Adotou-se o nível se confiança de 95% (Z^2^ 1-α/2 = 1,96), proporção esperada de 85% dos especialistas e um erro amostral de 15%, obtendo-se amostra ideal de 22 especialistas^(8)^. Considerando-se a dificuldade de retorno dos especialistas em estudos de validação, optou-se por convidar um número maior. Dessa forma, foram convidados 75 especialistas para participar como juízes na validação do conteúdo.

### Protocolo do estudo

Para identificar os elementos constitutivos do instrumento de avaliação da LC à pessoa com sífilis, desenvolveu-se uma Revisão de Escopo (RE), ou *scoping review*, na primeira etapa, conforme as seis etapas recomendadas: identificação das questões de pesquisa; identificação de estudos relevantes; seleção dos estudos; mapeamento e extração dos resultados; e síntese narrativa dos resultados^(9)^.

A elaboração da questão norteadora seguiu a estratégia intitulada de PCC (População - Conceito - Contexto) com a seguinte disposição: População - pessoa com sífilis; Conceito - avaliação da linha de cuidado; e, Contexto - dimensão Macropolítica e Micropolítica do cuidado em saúde. Deste modo, foi estabelecida a seguinte questão de pesquisa: Que elementos devem conter em um instrumento de avaliação da linha de cuidado à pessoa com sífilis na dimensão Macropolítica e Micropolítica do cuidado em saúde?

A busca foi realizada em janeiro a fevereiro de 2024 e dividida em três partes, com o intuito de identificar estudos primários publicados e não publicados (literatura cinzenta), bem como revisões. A primeira parte da busca ocorreu nas seguintes bases de dados: PubMed^®^, *Web of Sciense*, Scopus, CINAHL, *Cochrane Library, Scientific Electronic Library Online* e Literatura Latino-Americana e do Caribe em Ciências da Saúde a partir dos descritores indexados (Descritores em Ciências da Saúde - DeCS e *Medical Subject Headings - MeSH Database*), conjugados por meio dos operadores booleanos: Syphilis; *Health Evaluation;* Health Services; *Delivery of Health Care; Public Health Practice*; e *Health Services Administration*.

A segunda parte da busca ocorreu no *Google Scholar* ^®^, com uso dos termos índices e sinonímias (palavras-chave) identificados nos estudos da primeira parte da busca: “linha de cuidado”; “dimensão Macropolítica”; “dimensão Micropolítica”; “instrumento de avaliação”; “pessoa com sífilis”. A terceira parte da busca ocorreu mediante busca reversa, de acordo com as referências identificadas nas pesquisas selecionadas anteriormente. Essa estratégia de busca em três partes da scoping review permite a captação de outros materiais ou pesquisas não identificadas nas bases de dados^(9)^.

O processo de busca e seleção dos registros foi realizado por dois doutorandos de Enfermagem, munidos de protocolo específico, criado e registrado (*Open Science Framework* identificador (10.17605/OSF.IO/KPSAB) para este fim. Os impasses foram identificados e definidos através de consenso mútuo. A seleção dos estudos foi iniciada pela leitura flutuante (títulos e resumos) dos registros aplicando os critérios de elegibilidade, os registros aprovados foram encaminhados para a fase de leitura integral, a fim de compor a amostra final.

Os critérios de elegibilidade foram determinados a partir das questões de pesquisa, os quais incluiu-se estudos primários que abordassem elementos para construção de um instrumento de avaliação da linha de cuidado à pessoa com sífilis na dimensão Macropolítica e Micropolítica do cuidado em saúde, publicados em português, inglês ou espanhol, sem estabelecimento de temporalidade. A exclusão contemplou cartas ao editor, editoriais, artigos de revisão, estudos de casos únicos, resumos, anais, livros, protocolos, trabalhos de conclusão de curso, teses, dissertações e artigos não disponíveis na íntegra.

A coleta de dados foi orientada por protocolo original, os quais permitiu a conformação da tabulação de dados no *software* Microsoft Excel, versão 2013. Foram extraídas da amostra as seguintes informações: autor(es), ano de publicação, país de origem do estudo, idioma, periódico, área de escopo do periódico, base de dados, desenho do estudo, nível de evidência, objetivo do estudo, atributos, antecedentes, consequentes, outros conceitos e resultados do estudo. Com estes dados, possibilitou-se uma compreensão profunda e coesa dos estudos levantados que permitissem uma avaliação criteriosa.

Inicialmente, foram identificados 5.622 registros por meio da pesquisa nas bases. Após a remoção de duplicatas, os inelegíveis e os removidos por não atender aos critérios, 5.322 registros foram submetidos à triagem com a leitura dos títulos e resumos, resultando na exclusão de 5.329 registros. Em seguida, 115 artigos de texto completo foram avaliados para elegibilidade, dos quais 84 foram excluídos com base nos critérios predefinidos. Ao fim, foram analisados um total de 31 produções.

A partir disto, surgiram os temas de avaliação para composição do instrumento. Assim, foram definidas as seguintes unidades temáticas: Educomunicação; Qualificação de Informações Estratégicas; Ampliação dos Comitês de Investigação para Prevenção da Transmissão Vertical de HIV, Sífilis e Hepatites Virais.; Fortalecimento das Redes de Atenção à Saúde; Fortalecimento da parceria do MS com outros atores; Resposta Rápida à Sífilis nas Redes de Atenção à Saúde. Com isso, estruturou-se o instrumento mediante a categorização dos temas conforme a linha do cuidado segundo as perspectivas micro e macropolíticas de gestão^(10)^.

O processo de construção do instrumento consistiu, inicialmente, na definição dos itens que o compuseram, por meio da revisão de escopo, cuja análise foi norteada pelo referencial da LC na perspectiva da micropolítica e da macropolítica, além da Agenda Estratégica para Redução da Sífilis no Brasil^(11)^ e do Protocolo Clínico e Diretrizes Terapêuticas para Prevenção da Sífilis^(12)^. A partir dos resultados da revisão de escopo, os principais itens foram selecionados para compor o instrumento.

Dessa maneira, iniciou-se a construção preliminar do instrumento. Foram realizadas reuniões com quatro pesquisadores especialistas com conhecimento sobre sífilis para a análise das perguntas e respostas previamente elaboradas. Após a discussão, os itens foram reescritos ou mantidos conforme as recomendações de cada pesquisador, chegando-se a um consenso sobre o número mínimo de categorias selecionadas. A linguagem empregada foi direcionada a uma comunicação de fácil entendimento, clara, simples e objetiva, sem permitir ambiguidade na interpretação. Após o consenso entre os pesquisadores e os autores do instrumento, este ficou constituído por 74 itens.

Na terceira etapa, a validação de conteúdo, participaram profissionais de saúde, sendo utilizada a técnica Delphi, com necessidade de duas rodadas para atingir o consenso. O contato ocorreu via e-mail, com envio da carta-convite, TCLE e o link do formulário eletrônico disponibilizado no SurveyMonkey. Foi estabelecido o prazo de 20 dias para que cada avaliador aceitasse o TCLE e respondesse ao instrumento, a contar da data de envio do e-mail.

A aplicação dos formulários ocorreu no período de fevereiro a abril de 2024. Os convidados que não avaliaram o instrumento dentro do prazo estabelecido, foram excluídos da amostra. Foi solicitado aos juízes que avaliassem cada item de acordo com os critérios de clareza, compreensão, linguagem e relevância, a fim de verificar a dimensão e representatividade dos domínios do instrumento. Também, puderam sugerir a retirada, acréscimo ou modificações dos itens.

No instrumento construído, os profissionais especialistas emitiram concordância assinalando um “x” em uma escala do “tipo Likert” contendo: 1) Nada pertinente; 2) Pouco pertinente; 3) Pertinente e 4) Muito pertinente”. Assim, 29 especialistas participaram da primeira rodada e 22 na segunda rodada, sendo em sua maioria do sexo masculino, da área da docência e com titulação de doutorado.

Já na última etapa, houve a formatação do instrumento de coleta de dados em sua versão final. O instrumento novamente foi apresentado aos juízes para que reafirmassem e endossassem as alterações realizadas e, então, fosse formatada a versão final do instrumento. Todos os juízes aprovaram como versão a ser aplicada.

### Análise dos resultados

Os dados obtidos foram analisados a partir da avaliação das respostas dos formulários aplicados aos participantes do estudo. As informações coletadas foram armazenadas e processadas em banco de dados informatizados por meio dos softwares *Microsoft Office Excel* e *Statistical Package for the Social Sciences* (SPSS) versão 22.0.

Para a avaliação da consistência interna do instrumento (procedimentos analíticos), foram mensurados os Índices de Validade de Conteúdo aplicados aos Itens (IVC-I), de Fidedignidade aplicado aos Domínios (IFD) e de Validade de Conteúdo aplicado ao Instrumento (IVI)^(13)^, considerando-se a frequência simples das respostas dos especialistas. Desse modo, foram mantidos no instrumento (nas duas rodadas de avaliação) apenas os itens cujos valores percentuais de concordância foram iguais ou superiores a 80% (0,80).

Ao final de cada item, objeto da avaliação, havia um campo específico para respostas abertas, no qual os juízes especialistas poderiam justificar suas respostas e/ou descrever sugestões que contribuíssem para a pertinência do instrumento. O escore do índice foi calculado por meio da soma de concordância dos itens que foram marcados com (3) ou (4) pelos especialistas. A fórmula para avaliar cada item individualmente foi: IVC-I = número de respostas (3) + (4) / número total de respostas.

Ademais, para a análise de consistência e confiabilidade do instrumento, foi calculado o coeficiente de alpha de Cronbach, que é um dos mais importantes testes difundidos em pesquisas que envolvem a construção de testes e sua aplicação e que permitem verificar a correlação entre respostas de um questionário. Para análise do alpha de Cronbach, adotou-se o valor ≥ 0,70, que é considerado aceitável para estudos exploratórios e fornece evidências de uma escala internamente consistente.

## RESULTADOS

A versão inicial do instrumento possuía 74 questões, sendo a versão final estruturada com 58, sendo 49 na Dimensão 1 - Macropolítica do cuidado em saúde, e nove na Dimensão 2 - Micropolítica do cuidado em saúde.

O processo de validação de seu conteúdo foi realizado em duas rodadas (Delphi 1 e Delphi 2). Considerando as avaliações da rodada Delphi 1, algumas questões foram excluídas ou modificadas. Das questões contidas na primeira versão do instrumento, 16 (21,62%) não foram estabilizadas na primeira rodada e 18 (24,32%) tiveram sugestão de reformulação na escrita. Ao final da Delphi 2, foi obtido o IVC-I igual a 1,00 em cada item e o IVC de 1,00. Em seguida, foram realizados pequenos ajustes e o instrumento foi concluído com 58 itens, finalizando assim a etapa de validação de conteúdo. Quanto ao IVI, o instrumento pontuou 0,84 na Delphi 1 e 1,00 na Delphi 2.

Por meio da Alpha de Cronbach, verificou-se a confiabilidade dos dados. Pode-se observar que todos os domínios avaliados pelos especialistas obtiveram um alpha acima de 0,80. Do ponto de vista das dimensões, a fase 2 apresentou resultado melhor em termos gerais do que a fase 1, porém em ambas a confiabilidade foi bastante satisfatória, apresentada na Tabela 1.

**Tabela 1:** Confiabilidade dos dados em relação aos itens e dimensões do Instrumento de Avaliação da Linha de Cuidado à pessoa com Sífilis, Natal, Rio Grande do Norte, Brasil, 2024

Dimensões	Alpha de Cronbach	
	Fase 1	Fase 2
Dimensão 1- Macropolítica do cuidado em saúde	0,865	0,984
Dimensão 2- Micropolítica do cuidado em saúde	0,818	0,977
Avaliação geral	0,825	0,972

O Quadro 1 apresenta a versão final do Instrumento de Avaliação da LC à Pessoa com Sífilis, assim como o IVC-I de cada item e o IFD dos domínios.

**Quadro 1:** Instrumento de Avaliação da Linha de Cuidado à Pessoa com Sífilis - Versão Final, Natal, Rio Grande do Norte, Brasil, 2024

Dimensão 1- Macropolítica do cuidado em saúde		IVC-I	IFD
01	A Linha de Cuidado à Pessoa com Sífilis está inserida no Plano Estadual de Saúde?	1,00	1,00
02	A Linha de Cuidado à Pessoa com Sífilis organiza os fluxos e contrafluxos das pessoas na Atenção Primária à Saúde (APS), nos sistemas de apoio, nos pontos de atenção secundários e terciários?	1,00	
03	A Linha de Cuidado à Pessoa com Sífilis dispõe de protocolos clínicos e diretrizes terapêuticas, atualizados, à criança exposta à sífilis e sífilis congênita, à gestante com sífilis e sífilis adquirida?	1,00	
04	Os serviços de saúde operam com Sistemas de Informação para registrar, monitorar os indicadores de processo e disponibilizar informações clínicas, epidemiológicas e gerenciais sobre a sífilis?	1,00	
05	É realizada a elaboração e divulgação dos dados epidemiológicos da sífilis anualmente?	1,00	
06	É realizada a investigação e notificação de sífilis adquirida, congênita e em gestantes nos serviços de saúde, com segmento clínico laboratorial e encerramento dos casos?	1,00	
07	A Linha de Cuidado à Pessoa com Sífilis estabelece a integração entre os serviços especializados, centro de testagem e aconselhamento, hospitais, maternidades e APS?	1,00	1,00
08	A APS está sendo a porta de entrada principal para o cuidado às pessoas com sífilis, concentrando-se nas ações de promoção, prevenção, diagnóstico, tratamento e controle da sífilis?	1,00	
09	Na APS cada membro da equipe possui habilidades e competências definidas para o cuidado à pessoa com sífilis?	1,00	
10	A APS está organizada para oferecer os cuidados adequados às gestantes com sífilis e suas parcerias, conforme as recomendações preconizadas pelo Ministério da Saúde?	1,00	
11	A APS monitora as crianças expostas à sífilis e com sífilis congênita?	1,00	
12	A APS oferece testagem rápida para a sífilis durante todo o horário de funcionamento das unidades de saúde?	1,00	
13	A APS oferta teste não-treponêmico para o monitoramento após o tratamento (controle de cura) da sífilis?	1,00	
14	A APS administra a Benzilpenicilina benzatina?	1,00	
15	O referenciamento da APS para os outros serviços de saúde, contém informações da conduta prévia à qual a pessoa com sífilis foi submetida e aos resultados dos testes/exames?	1,00	
16	Há serviços ambulatoriais especializados de referência para pessoas com sífilis adquirida?	1,00	
17	As crianças com sífilis congênita têm acesso garantido, semestralmente, por 2 anos, à consulta oftalmológica?	1,00	
18	As crianças com sífilis congênita têm acesso garantido, semestralmente, por 2 anos, ao exame audiológico?	1,00	
19	As crianças com sífilis congênita têm acesso garantido, semestralmente, por 2 anos, à consulta neurológica?	1,00	
20	As crianças com sífilis congênita têm acesso garantido ao teste treponêmico (realizado a partir dos 18 meses de idade), quando necessário?	1,00	
21	As crianças com sífilis congênita têm acesso garantido ao hemograma, quando necessário?	1,00	
22	As crianças com sífilis congênita têm acesso garantido aos exames para avaliação de função hepática, pancreática, renal e distúrbios eletrolíticos quando necessário?	1,00	
23	Há serviços de atenção secundária para a realização da propedêutica e investigação para neurossífilis?	1,00	
24	Há serviços de atenção secundária para a realização da punção lombar, quando necessário?	1,00	
25	Há serviços de atenção secundária para a realização do tratamento da neurossífilis?	1,00	
26	Indivíduos tratados para neurossífilis têm o acesso à realização da punção lombar de controle?	1,00	
27	Há acesso para o tratamento para as gestantes diagnosticadas com sífilis na maternidade/casa de parto?	1,00	
28	Os recém-nascidos com diagnóstico de sífilis congênita têm acesso garantido na maternidade ao hemograma?	1,00	
29	Os recém-nascidos com diagnóstico de sífilis congênita têm acesso garantido na maternidade aos exames para avaliação de função hepática, pancreática, renal e distúrbios eletrolíticos?	1,00	
30	Os recém-nascidos com diagnóstico de sífilis congênita têm acesso garantido na maternidade ao exame de líquor - LCR?	1,00	
31	Os recém-nascidos com diagnóstico de sífilis congênita têm acesso garantido na maternidade a radiografia de ossos longos?	1,00	
32	Os recém-nascidos com diagnóstico de sífilis congênita têm acesso garantido na maternidade a radiografia de tórax?	1,00	
33	Há serviços na atenção terciária para o diagnóstico da neurossífilis?	1,00	
34	Há um instrumento de contrarreferência eficaz?	1,00	
35	O sistema de assistência farmacêutica contempla a medicação Benzilpenicilina benzatina para o tratamento de sífilis?	1,00	
36	O sistema de assistência farmacêutica contempla a medicação Benzilpenicilina procaína para o tratamento de sífilis congênita?	1,00	
37	O sistema de assistência farmacêutica contempla a medicação Benzilpenicilina cristalina (potássica) para o tratamento de neurossífilis?	1,00	
38	O sistema de assistência farmacêutica, nas unidades de saúde, sempre tem estoque suficiente de medicamentos para o tratamento das pessoas com sífilis?	1,00	
39	O sistema de assistência farmacêutica engloba a farmacovigilância no cuidado à sífilis?	1,00	
40	A gestão do cuidado engloba a Educação Permanente dos profissionais envolvidos com o cuidado à pessoa com sífilis?	1,00	1,00
41	Os momentos de Educação Permanente são realizados no próprio serviço, em tempo protegido (no horário de trabalho)?	1,00	
42	O processo de Educação Permanente ocorre por meio de metodologias ativas de ensino, aprendizagem significativa e crítica?	1,00	
43	O processo de Educação Permanente se dá ao longo de toda a vida institucional dos profissionais envolvidos no cuidado à pessoa com sífilis?	1,00	
44	Existe a integração ensino-serviço e comunidade?	1,00	
45	Os serviços que compõem a Linha de Cuidado à Pessoa com Sífilis disponibilizam preservativos como parte da rotina de atendimento?	1,00	
46	Elaboração e divulgação de materiais educativos e informativos sobre a prevenção, diagnóstico, tratamento e vigilância da sífilis para a população geral, populações-chave, populações prioritárias, entre outros?	1,00	
47	Existem parcerias entre gestores federais, estaduais e municipais, profissionais de saúde, comunidade e demais atores envolvidos na prevenção da sífilis?	1,00	
48	Os recursos financeiros estão disponíveis para a organização da Linha de Cuidado à Pessoa com Sífilis?	1,00	
49	Há o monitoramento e a avaliação da Linha de Cuidado à Pessoa com Sífilis?	1,00	
**Dimensão 2- Micropolítica do cuidado em saúde**		**IVC-I**	**IFD**
50	As equipes da APS reconhecem o seu papel no cuidado das pessoas com sífilis?	1,00	1,00
51	Nos serviços da APS os usuários são atendidos pelo(a) mesmo(a) médico(a) e/ou enfermeiro(a) ?	1,00	
52	A equipe da APS elabora o Projeto Terapêutico Singular em conjunto com o usuário com sífilis?	1,00	
53	Na APS, o prontuário clínico do usuário está disponível durante os atendimentos?	1,00	
54	Os profissionais da APS realizam a integração e inclusão da temática sífilis nos diferentes espaços do território (ex: escolas e associações) e nos diferentes espaços do serviço de saúde (ex: grupo de gestantes, grupo de idosos, na sala de espera, durante a consulta) ?	1,00	
55	A assistência farmacêutica monitora se o usuário completou o tratamento para sífilis?	1,00	
56	O sistema de assistência farmacêutica contempla a atenção farmacêutica à população com sífilis. Entende-se por atenção farmacêutica as ações realizadas por farmacêuticos para orientar e acompanhar o usuário do serviço de saúde quanto ao uso adequado dos medicamentos?	1,00	
57	Durante os momentos de reuniões, as equipes de saúde realizam o planejamento, monitoramento e avaliação do cuidado oferecido às pessoas com sífilis?	1,00	
58	A realização do planejamento, monitoramento e avaliação das ações de prevenção e controle da sífilis inclui a participação organizada da comunidade?	1,00	

*IVC-I - Índice de Validade de Conteúdo por Item; IFD - Índice de Fidedignidade do Domínio; APS - Atenção Primária à Saúde; LCR - Líquido Cefalorraquidiano.*

## DISCUSSÃO

O presente estudo elaborou um instrumento cuja proposta foi a avaliação criteriosa de diferentes aspectos que envolvem desde questões relacionadas à macropolítica quanto à micropolítica de saúde, que possibilite avaliar a operacionalização da LC à pessoa com sífilis. A sua aplicação poderá contribuir para o planejamento e aprimoramento das ações e serviços de atenção à sífilis, dentro da RAS. Poderá ser utilizado por gestores do SUS, por um grupo de profissionais de diferentes serviços da RAS e por pesquisadores de diversas instituições.

Apesar dos esforços governamentais dos últimos anos, o país continua distante da eliminação da sífilis congênita e do controle da sífilis adquirida e em gestantes. Um dos maiores desafios do SUS ainda é a fragmentação do cuidado, o que gera diversas barreiras ao acesso da população aos serviços de saúde. Na perspectiva da LC, essa fragmentação pode ocorrer em função de: ausência de ações de prevenção de doenças e agravos e práticas de promoção da saúde; ausência de gestão e regulação dos macroprocessos; deficiência da rede de serviços; desresponsabilização do cuidador; falta de insumos; qualidade questionável na prestação dos serviços; e dificuldade no acesso do usuário à assistência^(10)^.

Um sistema de saúde pautado pela integralidade deveria assegurar a continuidade da atenção, articulando um programa de cuidado responsável desde a APS, passando pelo ambulatório especializado até a urgência do hospital/maternidade^(14)^. Neste sentido, fazem-se urgentes iniciativas e novas tecnologias que possibilitem o aprimoramento da gestão do sistema de saúde na perspectiva da integração dos serviços, a partir de uma análise em nível micropolítico e macropolítico.

Diante da perspectiva da macropolítica, ou seja, da gestão, pensa-se em uma proposta de implantação da LC^(10)^. Para a sua efetivação, é necessária a organização da vigilância e da informação em saúde, comunicação em saúde, ações intersetoriais, implantação de políticas específicas com definição do financiamento, organização da rede de serviços, além da implantação de protocolos. Na perspectiva da micropolítica, ou seja, do processo de trabalho, os mesmos autores enfatizam o trabalho em equipe na coordenação do cuidado, a vinculação e responsabilização do cuidador, e a busca da autonomia do usuário.

Ainda em relação à macropolítica, primeira dimensão do instrumento do estudo, se propõe a realizar a organização e estruturação da LC no âmbito das políticas públicas de saúde, reforçando a importância de se incluir a LC à pessoa com sífilis no Plano Estadual de Saúde, uma vez que isso permite a integração dessa condição nas políticas de saúde e garante a articulação entre diferentes níveis de atenção. Ademais, ao se integrar os fluxos e contrafluxos entre os serviços de Atenção Primária, Secundária e Terciária se oportuniza que seja assegurado o seguimento adequado^(15)^.

No Brasil, a porta de entrada prioritária para o diagnóstico e tratamento da sífilis é a Atenção Primária à Saúde. Dessa forma, estes serviços precisam estar acessíveis, tanto geograficamente, quanto no que se refere à acessibilidade funcional, como horário de funcionamento, disponibilidade de teste rápido e antibiótico para o tratamento^(16)^. Além da construção do planejamento e realização do monitoramento contínuo das ações nos territórios, ter profissionais capacitados para o manejo adequado da sífilis, com escuta qualificada, sem julgamentos e preconceitos.

Os sistemas de informação em saúde são cruciais para monitorar a sífilis, embasando decisões em promoção, controle e transparência nas ações de vigilância^(17)^. Para gerar dados confiáveis, é essencial garantir qualidade e reduzir subnotificações, promovendo a conscientização dos profissionais de saúde^(18)^. Nesse sentido, a coleta e divulgação de dados, junto à notificação de casos, permitem ajustar estratégias locais e rastrear, tratar e prevenir a propagação da doença, com foco na notificação precoce para medidas imediatas de controle e seguimento clínico adequado^(19)^.

O manejo da sífilis em gestantes e suas parcerias torna-se essencial para a prevenção da sífilis congênita, contribuindo para a redução de complicações maternas e fetais e para evitar a reinfecção por meio de ações preventivas. A busca ativa de parcerias sexuais destas gestantes é uma estratégia relevante, pois favorece o diagnóstico, tratamento e o fortalecimento do pré-natal do parceiro nos serviços de saúde^(20)^.

É importante ressaltar que parceiros podem estar infectados mesmo com testes imunológicos não reagentes, exigindo tratamento adequado e reforçando a necessidade de capacitação dos profissionais de saúde para agir de forma eficaz nessas situações.

Um outro público foco de atenção no cuidado da APS é o das crianças, de modo que o Ministério da Saúde preconiza que todas as crianças expostas à Sífilis Congênita na gestação devem receber acompanhamento com consultas ambulatoriais mensais até o 6º mês de vida e bimestrais do 6º ao 18º mês, mesmo com mães adequadamente tratadas. Tal monitoramento tem como objetivo a identificação e o tratamento de possíveis casos de Sífilis Congênita. O seguimento adequado da Sífilis Congênita é fundamental para evitar complicações, sequelas tardias e garantir o desenvolvimento saudável das crianças^(21)^.

Torna-se importante destacar que o tratamento eficaz da sífilis envolve toda a RAS. O referenciamento da APS deve incluir informações completas para garantir continuidade e qualidade no cuidado^(22,23)^. Serviços especializados são essenciais para diagnóstico e manejo de complicações graves, como a neurossífilis, que exige atenção secundária devido à sua complexidade, sendo vital para o cuidado integral^(24)^.

Alguns municípios no Brasil, como Joinville, Paraná, São Paulo, Belo Horizonte e Rio de Janeiro, implantaram a Linha de Cuidado para pessoas com sífilis, mas enfrentam desafios na avaliação e monitoramento de sua efetividade. A diversidade das ações de controle e as diferenças regionais em um país de dimensões continentais dificultam a criação de instrumentos aplicáveis a todos os serviços e com validação de seu construto e aplicabilidade^(25)^. Contudo, a validação desses instrumentos pode contribuir para mudanças nos processos, qualificação dos serviços e implantação de modelos assistenciais alinhados às necessidades da população.

O estudo voltado à construção e validação de um instrumento de avaliação da linha de cuidado à pessoa com sífilis demonstra impactos significativos na prática da enfermagem, especialmente no âmbito da saúde pública. Entre os benefícios destacados, está o aprimoramento da prática clínica, possibilitando aos enfermeiros o uso de uma ferramenta objetiva para avaliar e monitorar a qualidade da assistência, facilitando a identificação de lacunas e a implementação de melhorias no cuidado prestado.

Além disso, o fortalecimento do papel estratégico dos enfermeiros como líderes no enfrentamento da sífilis é uma contribuição essencial. A aplicação do instrumento validado reforça ações integradas e resolutivas, evidenciando a importância desses profissionais na gestão de condições de saúde pública. A utilização de instrumentos validados também fomenta decisões fundamentadas, contribuindo para intervenções eficazes e personalizadas, otimizando os resultados para os pacientes.

Por fim, o estudo impacta diretamente a formação acadêmica e as investigações científicas, gerando conhecimento relevante que pode ser incorporado tanto no processo formativo de novos enfermeiros quanto em pesquisas futuras que visem ao aprimoramento contínuo da assistência à pessoa com sífilis.

### Limitações do estudo

Uma das limitações do estudo inclui a subjetividade dos juízes na interpretação das questões do instrumento. Acrescenta-se que como não foi realizada a validação do instrumento em cenários clínicos reais, pode não refletir plenamente os desafios e dinâmicas encontrados no dia a dia dos serviços de saúde.

### Contribuições para a área da enfermagem, saúde ou política pública

Esta nova ferramenta para a gestão do SUS poderá contribuir com a avaliação e o monitoramento dos serviços ofertados à comunidade, além de servir de base para a elaboração de ações estratégicas para o controle da sífilis, contribuindo para a melhoria do cuidado às pessoas com essa infecção nos serviços de saúde, na perspectiva da integralidade.

A enfermagem desempenha um papel fundamental na coordenação do cuidado e na implementação de práticas integradas que atendam às necessidades de pessoas com sífilis, conectando diretamente com o objetivo da pesquisa de construir e validar o instrumento de Avaliação da Linha de Cuidado. Esse instrumento oferece aos enfermeiros uma ferramenta robusta para monitorar a qualidade da assistência, identificar lacunas e promover melhorias. Além disso, ao alinhar a atuação estratégica da enfermagem na gestão e planejamento de políticas públicas, o estudo fortalece a base científica e prática para ações inovadoras que ampliem a eficácia do enfrentamento da sífilis.

## CONCLUSÕES

O Instrumento de Avaliação da Linha de Cuidado à Pessoa com Sífilis, validado por especialistas, apresenta 58 questões distribuídas nas dimensões macropolítica e micropolítica do cuidado em saúde, garantindo parâmetros de validade satisfatórios. Sua aplicação na avaliação dos serviços de saúde pode gerar melhorias na acessibilidade dos usuários e na integração entre serviços e profissionais, fortalecendo o cuidado à pessoa com sífilis, demonstrando a relevância da pesquisa.

Espera-se que, com uma Linha de Cuidado de qualidade, as taxas de transmissão sejam reduzidas, promovendo mudanças significativas no perfil epidemiológico no Brasil.

## Data Availability

Os dados da pesquisa estão disponíveis no corpo do artigo.
